# Serious non-fatal unintentional injuries among in-school adolescents in Sri Lanka: results from the 2016 Sri Lankan global school-based health survey

**DOI:** 10.1186/s12889-020-09839-3

**Published:** 2020-11-12

**Authors:** Sashimali Wickramasinghe, Nalika Sepali Gunawardena, Dhanusha Punyadasa, Shanthi Gunawardena, Champika Wickramasinghe, Ayesha Lokubalasooriya, Renuka Peiris, Sameera Senanayake

**Affiliations:** 1 Ministry of Health, Colombo, Sri Lanka; 2 World Health Organization, Country Office, Colombo, Sri Lanka; 3 Ministry of Education, Pelawatta, Sri Lanka; 4 Queensland University of Technology (QUT), Brisbane, Australia

**Keywords:** Unintentional injuries, Adolescents, Sri Lanka

## Abstract

**Background:**

Unintentional injuries among adolescents is a major public health problem the world over. A great majority of the annual deaths among adolescents is due to unintentional injuries; it is also the leading cause of death among adolescents in the world. The aim of this study was to estimate the incidence of injuries and their associated factors among school going adolescents aged 13–17 years using data from the most recent Global School-Based Health Survey (GSHS) conducted in Sri Lanka.

**Methods:**

A cross-sectional survey was conducted using a self-administered questionnaire, among 3262 adolescents attending government schools. The sample was selected through a two-staged cluster sampling technique. In the first stage, 40 schools were selected using probabilities proportional to school enrollment size, from all schools in the country that have classes in grades 8–12. Then, from the selected schools, classes were selected using systematic equal probability sampling with a random start. The weighted prevalence was calculated, and logistic regression analysis was conducted in order to determine the correlates.

**Results:**

During the 12 months before the survey, 35.8% (95% CI-30.7 - 41.1) of the students reported being seriously injured one or more times. The injuries were more common among males, but were equally common among the two different age groups (13–15 age group vs 16–17 age group). The most common type of injury was cut or stab wounds (5.5%), followed by broken bones/dislocated joints (5.3%). Multivariable analysis revealed that only a few factors were associated with injury, such as being of the male sex, being bullied, being physically attacked, and/or being in a physical fight.

**Conclusion:**

This study demonstrated that the prevalence of serious unintentional injuries among school going adolescents is a major public health problem in Sri Lanka. This timely and comprehensive survey would help policy makers and researchers identify the unmet needs related to adolescent injuries. Furthermore, evidence generated form the study should be given due consideration when designing school-based interventions to prevent adolescent injuries.

## Background

Adolescents are vulnerable to multifarious injuries because of their size, growth and development, inexperience, and natural curiosity [1]. These injuries can occur either intentionally or unintentionally. Intentionality distinguishes violence from unintended events that result in injury [1]. Unintentional injuries among adolescents is a major public health crisis the world over. Interestingly, more than 95% of injury related deaths among adolescents occur in low and middle-income countries (LMIC) [2]. Analysis of data from 68 LMICs indicated that the overall prevalence of unintentional injuries among adolescents in the 13–15 year age group during the past 12 months is 42.9% (95% CI 39.0–46.9) [3]. Furthermore, the leading cause of death among adolescents aged 15–19 years is unintentional injuries [4].

Evidence indicates that for every injured adolescent who dies, there are several thousand adolescents who live on with varying degrees of disability [5]. Furthermore, significant economic costs and lifelong social burdens are caused by non-fatal adolescent injuries [6]. Considering the type of unintentional injuries, the five most common injuries in adolescents are road traffic injuries, falls, burns, drowning, and poisoning [7, 8]. These five categories make up 60% of deaths due to adolescent injury worldwide [2]. Other injuries that could occur are open wound injuries, fractures, and muscle and joint injuries [9].

In developed countries, unintentional injuries have replaced infectious diseases as the most serious public health problem among adolescents [1, 10]. However, the true extent of the problem in developing countries is largely unknown owing to the lack of national level surveys. Therefore, information is urgently needed on the incidence rates as well as the factors contributing to adolescent unintentional injuries in developing countries.

Similar to many developing countries, there is a scarcity of knowledge in this regard in Sri Lanka. Most studies on adolescent injuries conducted in Sri Lanka are hospital-based studies. Injuries necessitating hospitalization are relatively small in number, and hospital data is only the tip of the iceberg [2]. Therefore, national level community-based studies are essential to identify the true magnitude of the problem in addition to prioritizing preventive strategies.

The Global School-Based Student Health Survey (GSHS), which was initiated in 2001 as a collaborative effort of the World Health Organization (WHO), The United Nations Joint Programme on HIV/AIDS (UNAIDS), The United Nations Educational, Scientific and Cultural Organization (UNESCO), and the United Nations Children’s Fund (UNICEF) along with technical assistance from the US Center for Disease Control and Prevention (CDC), is a rich source of information on health and related information on school going adolescents [11].

Adolescents consist of one fifth of the population of Sri Lanka. Among the estimated number of 3.8 million adolescents in the country, close to 70% of adolescents attend school [12]. The GSHS was first conducted in Sri Lanka in 2008 among school going adolescents, and it provided valuable information on their health behaviors. This study revealed that among students in the 13–15 year age group, 37.2% (95% CI-32. 1–42.2) have had at least one serious injury in the previous year. Obtaining a clear view of the burden of the issue necessitates detailed information on the magnitude, trends, and influencing factors of the problem. Furthermore, repeated GSHSs are recommended by the WHO to assess the trends in the prevalence of risk factors [13].

Thus, the aim of this paper is to estimate the incidence of injuries and their associated factors among school going adolescents aged 13–17 years using the data of the most recent GSHS conducted in Sri Lanka. This is the first national survey conducted among school going adolescents since the first GSHS conducted in 2008. Therefore, the information on the extent of adolescent unintentional injuries and associated factors in local settings gathered from this survey would be useful for health policy makers in the formulation of injury prevention policies and a strategic framework. It could also be useful for integrating injury preventive interventions into the existing school health programme in Sri Lanka.

## Methods

This descriptive cross-sectional study involved the analysis of existing data from the Sri Lankan Global School-Based Health Survey conducted in 2016. The GSHS intends to provide data on health behaviors and associated risk and protective factors among school going adolescents. Students of grades 8, 9, 10, 11 and 12 in government schools (which corresponds to the 13 to 17 year age group) in Sri Lanka were recruited for the survey. Data collection was done during the period 1st October 2016 to 31st November 2016. The methodology developed by the WHO and the US Center for Disease Control and Prevention (CDC) has been adopted in the current study [14].

### Sample size and sampling

A sample of 3125 students was deemed adequate to assess the magnitude of injuries in this group with a precision of ±5% and an 80% response rate. The sample was selected through a two-stage cluster sampling technique. In the first stage, 40 schools were selected, using probabilities proportional to school enrollment size, from all schools in the country that have grades 8–12 classes. Then, from the selected schools, classes were selected using systematic equal probability sampling with a random start. All students in the selected classes were invited to participate in the survey.

### The questionnaire

The GSHS questionnaire was adapted to Sri Lanka and was translated into Sinhala and Tamil. This was a self-administered questionnaire. Three questions were included in the questionnaire to obtain information relevant to injuries. The first question assessed the number of times the student was seriously injured during the past 12 months; here, seriousness was defined as ‘making the respondent miss at least one full day of usual activities (such as school, sports, or a job) or necessitating treatment by a doctor or nurse’. It had eight responses, with the frequency ranging from 0 to 12 or more times. The next question inquired about the most serious injury that had happened to the respondent during the 12 months prior to the survey. The measurement scale had the following responses: I was not seriously injured during the past 12 months; I had a broken bone or a dislocated joint; I had a cut or stab wound; I had a concussion or other head or neck injury, was knocked out, or could not breathe; I had a gunshot wound; I had a bad burn; I was poisoned or took too much of a drug; Something else happened to me. The final question looked into the cause of the most serious injury that had happened during the past 12 months. The responses included; I was not seriously injured during the past 12 months; I was involved in a motor vehicle accident or hit by a motor vehicle; I fell; Something fell on me or hit me; I was attacked or abused or was fighting with someone; I was in a fire or too near a flame or something hot; I inhaled or swallowed something bad for me; Something else caused my injury. Information required to assess the associations (socio demographic details, details of students’ mental health, substance use, and parental engagement in students’ life) were collected. Measures were taken to minimize any interference with school activities during data collection.

Relevant administrative clearance to conduct the study was obtained from the Ministry of Education, zonal education directors, and school principals. Ethical clearance to conduct the study was received from the Faculty of Medicine, University of Colombo.

### Data analysis

The SPSS version 21.0 was used for data analysis. The data set was cleaned and edited for inconsistencies. Missing data were not statistically imputed. The following weight (W) was assigned to each questionnaire in order to allow for differing patterns of non-response and the likelihood of sampling each student:

$$ W=W1\ast W2\ast f1\ast f2\ast f3 $$

W1 = Inverse probability of the school being selected.

W2 = Inverse probability of the classroom being selected within the school.

f1 = Non-response adjustment factor (i.e. school level) estimated based on the school size (small, medium, large).

f2 = Non-response adjustment factor (i.e. student level) estimated by class.

f3 = Adjustment factor estimated by grade, after post-stratification.

Incidences of injuries are presented in terms of having sustained serious injuries, and were reported using frequencies with 95% confidence intervals. The correlates of sustaining a serious injury were identified by conducting a bivariate analysis and a backward logistic regression analysis. The dependent variable was sustaining a serious injury within 12 months prior to the survey at least once or not. The independent variables were all categorical in type and included age, sex, being bullied, considering suicide, attempting suicide, feeling lonely most of the time, current substance use (alcohol, cigarettes, recreational drugs etc.), not having close friends, missing classes without permission, parents not understanding students’ problems, parents not knowing what students were doing during their free time, not being taught anger management in school, being in a physical fight, and being physically attacked. Except for age and sex, all other variables had more than one response and were dichotomized by categorizing any positive response as “Yes” and any negative response as “No”. Variables that resulted in a *p* value of 0.05 or less in the bivariate analysis were included in the backward multivariable logistic regression. Adjusted odds ratios with 95% confidence intervals were obtained for the significant associates identified by the logistic regression.

## Results

Among the 3650 students who were sampled, 3263 (89%) completed the questionnaires. However, only 3262 questionnaires were usable for the final analysis. Of the sampled population, 387 (3650 minus 3263) did not participate in the study. The reasons included not giving consent (*n* = 116) and being absent from school on the day of data collection (*n* = 271).

### Characteristics of the study sample

The majority of respondents were 13–15 years old (66.5%,), and females comprised 51.1% of the sampled population (Table 1).

**Table 1:** Description of study participants of the 2016 Sri Lankan Global School-Based Survey (including the variables used to assess the correlates of unintended injuries)

Characteristic	Number^a^	Percentage^b^
Sex (*n* = 3262)		
Male	1437	48.9
Female	1805	51.1
Age (*n* = 3261)		
< 13 years	66	2.1
13–15 years	2196	66.5
16–17 years	977	30.7
> 17 years	22	0.7
Was Bullied (*n* = 3196; missing =66)	1208	38.6
Felt lonely most of the time (*n* = 3250; missing =12)	294	9.1
Considered suicide (*n* = 3221; missing =41)	298	9.4
Current smoking (*n* = 3244; missing =18)	107	3.6
Current use of alcohol (*n* = 3191; missing =71)	103	3.3
Current use of recreational drugs (*n* = 3217; missing =45)	79	2.7
Had no close friends (*n* = 3245; missing =17)	180	5.6
Lacked parental supervision (*n* = 3233; missing =29)	987	30.8

^a^Unweighted frequency; ^b^Weighted percentage

During the 12 months before the survey, 35.6% (95% CI-30.7 - 41.1) of the students reported being seriously injured one or more times. Almost one out of two boys (45.3, 95% CI 39.7–51.1) had been seriously injured as compared to only one fourth of girls (26.4%), which was statistically significant. Serious injuries were equally common among students in younger (13–15 years) age groups and older (16–17 years) age groups (*p* > 0.05) (Table 2).

**Table 2:** Incidence of unintentional injuries among study participants (*N* = 3262)

	Was seriously injured at least once, within 12 months prior to survey % (95% CI)
**Total**	35.6 (30.7–41.1)
**Sex**	
Males	45.3 (39.7–51.1)
Females	26.4 (21.6–32.2)
**Age**	
13–15 years	35.9 (30.3–41.9)
16–17 years	34.8 (29.1–40.9)

The most common type of injury was a cut or stab wound (5.5%), followed by a broken bone/dislocated joint (5.3%) and a head injury or a concussion (1.2%). While the cut or stab wounds were the commonest injury type among the younger age group, broken bones/ dislocated joints were the commonest injury type among the older age group. Broken bones/ dislocated joints were the commonest injury type among males, while among females, the commonest injury type was cut or stab wounds (Table 3).

**Table 3:** Description of the serious injuries that has happened to the study participants

Type of injury	Age group				Sex				Total (***N*** = 3262)	
	13–15 years (***N*** = 2196)		16–17 years (***N*** = 977)		Male (***N*** = 1437)		Female (***N*** = 1805)			
	N	%	N	%	N	%	N	%	N	%
Cut or stab wound	134	6.1	41	4.2	113	7.9	65	3.6	181	5.5
Broken bone/ dislocated joint	121	5.5	49	5.0	116	8.1	57	3.2	173	5.3
Concussion/ Head injury	34	1.5	6	0.6	22	1.5	16	0.9	40	1.2
Burns	19	0.9	1	0.1	7	0.5	13	0.7	22	0.7
Poisoned	5	0.2	4	0.4	6	0.4	6	0.3	12	0.4
Something else	229	10.4	139	14.2	205	14.3	176	9.8	381	11.7

Analysis of the mechanism of the most serious injury that had happened during the past 12 months revealed that falls were the most common mechanism (12.1%), followed by motor vehicle crashes (3.0%) and something falling on the person (2.7%). Falls were the most common mechanism for injuries among both age groups and both sexes (Table 4).

**Table 4:** Description of the serious injuries that has happened to the study participants

Type of injury	Age group				Sex				Total (***N*** = 3262)	
	13–15 years (***N*** = 2196)		16–17 years (***N*** = 977)		Male (***N*** = 1437)		Female (***N*** = 1805)			
	N	%	N	%	N	%	N	%	N	%
Falls	293	13.3	95	9.7	223	15.5	168	9.3	395	12.1
Motor vehicle accidents	53	2.4	39	4.0	74	5.1	24	1.3	99	3.0
Something falling on the person	61	2.8	22	2.3	59	4.1	28	1.6	87	2.7
Attacks	14	0.6	8	0.8	13	0.9	9	0.5	22	0.7
Fire	7	0.3	1	0.1	6	0.4	3	0.2	9	0.3
Breathed a noxious material	8	0.4	3	0.3	4	0.3	9	0.5	14	0.4
Something else	119	5.4	66	6.8	91	6.3	97	5.4	190	5.8

### Factors associated with violence and injury

Bivariate analysis revealed that being of the male sex, being bullied, feeling lonely most of the time within the past 12 months, considering suicide, smoking, alcohol use, use of recreational drugs, being physically attacked or being in a physical fight, and lack of parental supervision were significantly associated with being seriously injured at least once in the year prior to the survey. Multivariable analysis revealed that only a few factors were associated with injury, namely, being of the male sex, being bullied, being physically attacked, and/or being in a physical fight (Table 5).

**Table 5:** Factors associated with being physically attacked (results of bi-variable and multi-variable analysis)

Characteristic		Bi-variable analysis OR (95%CI)	Multi-variable analysis aOR (95%CI)
Age	13–15 yrs	1.0 (0.8–1.2)^*^	–
	16–17 yrs	1	
Sex	Male	2.3 (1.9–2.7)	1.8 (1.5–2. 1)
	Female	1	1
Was Bullied	Yes	4. 1 (3.5–4.8)	2.3 (1.9–2.5)
	No	1	1
Felt lonely	Yes	2. 1 (1.6–2.7)	–
	No	1	
Considered suicide	Yes	2.2 (1.7–2.8)	–
	No	1	
Current smoking	Yes	3.8 (2.5–5.8)	–
	No	1	
Current use of alcohol	Yes	3.2 (2.1–4.9)	–
	No	1	
Current use of recreational drugs	Yes	7.7 (4.3–13.6)	–
	No	1	
Had close friends	No	1.3 (0.9–1.8)^*^	–
	Yes	1	
Lacked parental supervision	Yes	1.6 (1. 1–1.9)	–
	No	1	
Was physically attacked	Yes	3.5 (3.0–4. 1)	1.6 (1.3–1.9)
	No	1	1
Was in a fight	Yes	3.5 (3.0–4. 1)	1.9 (1.6–2.3)
	No	1	1

^*^Not significant at p = 0.05 level

## Discussion

Adolescents are more prone to injuries due to risk taking and sensation seeking behaviors [15]. This first national survey conducted among school going adolescents after the GSHS-2008 indicated that the common finding in the study group includes serious injuries necessitating medical attention and missing out daily activities. Approximately one in three children in the 13–17 year age group was seriously injured at least once within the 12 months prior to the survey. The prevalence of unintentional injuries has not changed over the years. A similar prevalence (37.2%) was found in the GSHS-2008 among 13–15 year olds [16], which indicates the necessity for novel public health approaches to combat this serious menace.

Unintentional injuries are a major contributor to adolescent mortality and morbidity. Direct comparison of results between countries and even within the same country, across different studies, is difficult due methodological differences (e.g. age of the study population). However, the comparison of GSHS studies provides useful information as they have used uniform definitions and study populations. Han et al. (2019) conducted a secondary analysis of GSHS data from 68 LMIC countries. The overall incidence of serious injuries among 12–15 year old adolescents was 42.9% [3], which means that roughly one in two adolescents has been seriously injured during the past 12 months. Similar to other LMIC countries, our study indicates that adolescent non-fatal injuries have the capacity to affect the daily activities of school going adolescents.

The current study was conducted as a community-based study, which is able to paint a more comprehensive picture of injuries when compared to hospital based studies [17, 18]. This is best described as an injury pyramid based on the level of medical treatment of injury victims [19]. The apex of the pyramid represents the injuries necessitating hospitalization, which are relatively small in number, whereas lower parts of the pyramid represent the more numerous injuries, which are of lesser severity. Since hospital-based studies can identify injuries that required hospitalization, they will only address the apex of the injury pyramid, which is the tip of the iceberg. Therefore, it is imperative to conduct community-based studies on adolescents’ unintentional injuries to identify the true magnitude of the problem.

A major limitation in comparing incidence data of unintentional injuries across studies is the lack of consistency in their assessment, recall periods, and definitions. It has been found that there is a wide variation in the incidence of injuries with varying recall periods. The results of studies using diverse recall periods cannot be compared without bearing in mind the factors affecting such differences [20]. However, since GSHSs have been conducted in several countries and they have all used a consistent definition and a recall period, it enables meaningful comparison across countries. Analysis of GSHS data of 13 to 15 year old school students of Indonesia, Myanmar, Sri Lanka and Thailand for the purpose of identifying the burden of serious injuries among school students has reported an overall prevalence of 42.2% for all countries, ranging from 27.0% in Myanmar to 46.8% in Thailand [21]. These figures indicate that the prevalence among low and middle-income countries is uniform across countries. However, relatively low figures have been reported from developed countries [9, 22].

Boys are more prone to serious injuries that necessitate medical attention and/or lead to missing out daily activities, which fact was corroborated by the current study. The male gender was found to be significantly associated with the occurrence of unintentional injuries in the present study. This was in line with some other studies which had shown similar sex differences in injury rates [22–24]. This may be explained by the fact that males have a higher probability of risk taking behavior, are physically more active, and have more probability of engaging in competitive sports [25, 26]. Several studies have demonstrated that older adolescents are more prone to unintentional injuries [27, 28]. However, similar to the GSHS results of 2008, the present study has failed to demonstrate this association.

The current study found that falls and motor vehicle accidents are the most common causes of unintentional injuries among school going adolescents, and similar studies have found these two to be the most common etiologies for the injuries [21, 29, 30]. Similarly, a large-scale household survey conducted to assess the epidemiology of unintentional injuries in Nepal has reported falls as the commonest cause for unintentional injuries among adolescents [31]. Likewise, vehicle accidents are also a leading cause of non-fatal injuries among adolescents [32]. Evidence indicates that vehicle accidents among adolescents cause a sizeable economic burden due to loss of productivity and medical costs [33]. Furthermore, it has been shown that the commonest vehicle involved in these accidents is the motor cycle, and most of the injured children were not wearing helmets at the time of injury [29]. Riding motor cycles without a helmet is common among adolescents in Sri Lanka, which points to a serious public health problem among adolescents that needs urgent attention [34]. This study has also reported that fatal road traffic accidents are more commonly seen among boys than among girls.

Though physical attacks have contributed to only 2.7% of all injuries, being in a fight was significantly associated with being injured. Globally, violence leads to 5.8% of injury related deaths among children below 17 years, and is more commonly seen among older adolescent groups (2). Therefore, more attention should be given to interpersonal violence among school children.

In addition to ill health, serious injuries also have dire effects on school performance. Thus, proper attention should be given to the provision of remedial measures for this problem. Knowledge about the extent and correlates of the problem is a valuable asset while designing remedial measures. According to the current study, being of the male sex, being bullied, being physically attacked and/or being in a physical fight were associated with suffering serious injuries. Identification of such vulnerable populations should be targeted while planning interventions for injury prevention.

This study has several limitations. Firstly, as the study was conducted among school going adolescents, generalizing the findings to the entire adolescent population in Sri Lanka is not possible, as the circumstances of the various adolescents may differ significantly. Secondly, the standard questionnaire used in the study did not have the geographical location of the injury. This could have been an important piece of knowledge with which to plan the intervention. Thirdly, the study population was school-going adolescents between 13 and 17 years, and it therefore does not represent the total school-going adolescent population, as, according to the WHO, an adolescent is defined as an individual between 10 and 19 years of age. Fourthly, the current study only included adolescents attending government schools, excluding those who attend private schools in the country. However, since private school attendees comprise only 3% of the school going population [35], the authors believe that the current sample adequately represents the total school going population. Finally, recall bias is a possible limitation, as information was solicited regarding the 12 months prior to the survey, and participants may not have remembered the information accurately.

## Conclusion

This study demonstrated that the prevalence of serious unintentional injuries among school going adolescents has not changed over the years, indicating that it is still a major public health problem in Sri Lanka. Health policy and interventions related to the prevention of adolescent serious non-fatal injuries require solid evidence. This timely and comprehensive survey would help policy makers and researchers identify the unmet needs in this sphere. School-based interventions to address these issues should be designed with the goal of increasing adolescents’ awareness of possible preventive measures related to unintentional injuries.

## Data Availability

The dataset used during the current study is available from WHO’s NCD Microdata Repository (https://extranet.who.int/ncdsmicrodata/index.php/catalog/648).

## Abbreviations

CDC: US Centres for Disease Control and Prevention
GSHS: Global School-based Student Health Survey
UNAIDS: United Nations Joint Programme on HIV/AIDS
UNESCO: United Nations Educational, Scientific and Cultural Organization
UNICEF: United Nations Children’s Fund
WHO: World Health Organization
YLDs: Years Lost due to Disability
